# Tumor Microenvironment‐Responsive Nanomedicines for Potentiating Cancer Immunotherapy

**DOI:** 10.1002/advs.202513567

**Published:** 2025-09-12

**Authors:** Qianqian Huang, Fan Tong, Jiantao Chen, Muzaffar Kayumov, Yue Lv, Yajun Shi, Bengui Ye, Huile Gao

**Affiliations:** ^1^ Shaanxi Province Key Laboratory of New Drugs and Chinese Medicine Foundation Research, Pharmacy College Shaanxi University of Chinese Medicine Xianyang 712046 China; ^2^ Key Laboratory of Drug‐Targeting and Drug Delivery System of the Education Ministry Key Laboratory of Leather Chemistry and Engineering (Ministry of Education) West China School of Pharmacy Sichuan University Chengdu 610041 China; ^3^ Medical College of Tibet University Lasa 850002 China; ^4^ Tianfu Jincheng Laboratory Chengdu 610093 China; ^5^ Institute of Bioorganic Chemistry of Uzbekistan Academy of Sciences Tashkent 100125 Uzbekistan

**Keywords:** cancer immunotherapy, nanomaterials, spatiotemporal control, tumor immune cycle, tumor microenvironment response

## Abstract

Immunotherapy has emerged as a transformative paradigm in oncology, yet its clinical efficacy remains constrained by the immunosuppressive tumor microenvironment and inadequate spatiotemporal control of existing modalities, resulting in suboptimal patient response rates and frequent immune‐related adverse events. Stimuli‐responsive biomaterials, capable of precise modulation over the delivery kinetics of immunotherapeutic agents, are validated as powerful strategies for achieving targeted immunomodulation. This review systematically delineates innovative design frameworks for tumor microenvironment (TME)‐responsive nanomaterials leveraging dynamic TME features. Through detailed dissection of cancer‐immunity cycle barriers, including spanning impaired antigen presentation, compromised T cell priming/activation, and defective effector function, breakthrough advances in next‐generation delivery platforms are highlighted that reignite antitumor immunity at a critical juncture. Furthermore, preclinical validation and clinical translation challenges of TME‐responsive nanomaterials are evaluated. Building upon current immunotherapy trends, this review also identifies critical translational determinants, with the aim of bridging the bench‐to‐bedside gap in precision cancer immunotherapy.

## Graphical abstract

Strategies for TME‐responsive Nanomaterials to Potentiate Cancer Immunotherapy.

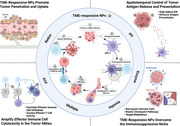

## 1 Introduction

Cancer immunotherapy, which harnesses the immune system to combat tumors, has evolved from a theoretical concept into a clinical reality over recent years, profoundly transforming the oncology therapeutic landscape.^[^ ^1^ ^]^ Landmark breakthroughs, notably immune checkpoint inhibitors,^[^ ^2^ ^]^ adoptive cell therapies,^[^ ^3^ ^]^ and therapeutic cancer vaccines,^[^ ^4^ ^]^ have achieved unprecedented durable responses in refractory malignancies. However, these advances remain challenged by core limitations: primary or acquired resistance, immune‐related adverse events, and the persistent barrier posed by the immunosuppressive tumor microenvironment (TME), all constraining therapeutic efficacy.^[^ ^5^ ^]^ Furthermore, heterogeneous tumor immunogenicity, insufficient T cell infiltration, and dynamic immune evasion mechanisms contribute to disease‐specific variations in treatment outcomes.^[^ ^6^ ^]^ Consequently, there is an urgent need to develop novel strategies targeting resistance pathways to overcome current limitations and advance the establishment of precision immunotherapy frameworks.

Tumor cells evade immune surveillance through multifaceted immunosuppressive mechanisms, contributing to resistance to immunotherapy. The TME critically shapes immune contexture and fate. Beyond T cells, innate immune cells and cancer‐associated fibroblasts (CAFs) generate fibrotic stroma that impairs T cell function and infiltration.^[^ ^7^ ^]^ At the cellular level, immunosuppressive populations expand aberrantly within the TME, inhibiting effector T cell function.^[^ ^8^ ^]^ Molecularly, the overexpression of immune checkpoints such as programmed cell death ligand 1 (PD‐L1/PD‐1)^[^ ^9^ ^]^ and cytotoxic T lymphocyte associated protein 4 (CTLA‐4),^[^ ^10^ ^]^ coupled with immunosuppressive signaling cascades driven by factors like indoleamine 2,3‐dioxygenase (IDO), transforming growth factor‐beta (TGF‐β), and vascular endothelial growth factor (VEGF),^[^ ^11^ ^]^ collectively drive T cell exhaustion. Physical barriers, including stromal fibrosis and aberrant vascular networks, hinder lymphocyte infiltration, creating immune‐excluded niches.^[^ ^12^ ^]^ Compounding this, tumor cells competitively deplete glucose and release lactate, leading to microenvironmental acidosis and adenosine triphosphate (ATP) deprivation, a combination that directly suppress cytotoxic T lymphocyte (CTL) killing capacity.^[^ ^13^ ^]^ These dynamically intertwined inhibitory networks exhibit spatiotemporal heterogeneity and undergo adaptive evolution via epigenetic reprogramming, ultimately undermining the integrity of the antitumor immune response.

Bioengineered strategies offer high tunability, enabling precise modulation of cancer immunotherapy to potentiate antitumor immune responses.^[^ ^14^ ^]^ TME‐responsive nanomedicines achieve spatiotemporal control over immunotherapeutic activity across the cancer‐immunity cycle by incorporating stimulus‐responsive elements into nanoparticles (NPs). These elements confer responsiveness to endogenous TME signals, including pH variations, redox potential gradients, hypoxia, overexpressed enzymes, and ATP levels.^[^ ^1^, ^15^ ^]^ Leveraging their dynamic sensing and precise modulation capabilities, these engineered TME‐responsive nanomedicines systematically potentiate immunotherapeutic efficacy at each stage of the cancer‐immunity cycle.

In this review, we comprehensively delineate the critical microenvironmental barriers impairing tumor immune recognition. Focusing on the intersection of materials science and immunology, we summarize strategies for designing responsive nanomaterial systems tailored to distinct TME features. Departing from conventional material‐centric classification, we present an innovative analysis framework structured around the cancer‐immunity cycle. We elaborate on emerging responsive material design strategies explored within each phase covering initiation, activation, and effector stage, aimed at refining and potentiating biomaterial‐based cancer immunotherapies. Furthermore, we critically evaluate the clinical translation potential of these approaches. Integrating current trajectories in immunotherapy development, we probe the pivotal considerations for translating these strategies from bench to bedside and provide forward‐looking perspectives on future directions in tumor immunotherapy.

## 2 Design Principles of TME‐Responsive Nanomaterials

The TME is characterized by low pH, elevated glutathione (GSH) levels, reductive conditions, hypoxia, and overexpression of enzymes and ATP.^[^ ^16^ ^]^ TME‐responsive nanomaterials integrate recognition units for these TME‐specific stimuli to construct multi‐modal sensing systems with dynamically tunable response thresholds.^[^ ^1^, ^15^, ^17^ ^]^ In their design, TME‐responsive nanomaterials employ pH‐sensitive bonds, reduction‐sensitive bonds, hypoxia‐responsive linkers, and enzyme‐cleavable peptides to achieve cascade amplification and functional transformation of stimulus‐response signals. This enables programmed manipulation of material structures, including targeted bond cleavage, NP disassembly, hydrogel swelling, and pore modulation. Ultimately, this programmed structural control in TME‐responsive nanomaterials facilitates precise drug release and activation of therapeutic functions, providing an efficient and controllable technological platform for precision tumor therapy.

### 2.1 pH‐Responsive Nanomaterials

The TME exhibits an acidic pH. The acidic microenvironment of tumors arises from distinct biological mechanisms that collectively establish a low pH. Spatial variations in acidity are evident, with most neoplastic tissues exhibiting a pH ranging from 6.5 to 7.0,^[^ ^18^ ^]^ while the underlying causes of this acidic condition involve multiple contributing factors. First, the heightened metabolic demand of rapidly proliferating cancer cells drives the production of acidic byproducts. Oxidative phosphorylation generates CO_2_, while glycolysis directly produces H⁺ ions. The Warburg effect further exacerbates acidity by promoting glycolysis even under aerobic conditions, resulting in significant lactate accumulation.^[^ ^19^ ^]^ Additionally, vascular dysfunction in certain tumors impairs the removal of these acidic metabolites, sustaining a low‐pH environment.^[^ ^20^ ^]^ Finally, an active proton‐extrusion mechanism in cancer cells plays a crucial role. These cells upregulate membrane transporters to expel H⁺ ions into the extracellular space, thereby preserving intracellular pH at ≈7.4—slightly alkaline compared to the physiological value of 7.2.^[^ ^21^ ^]^ This intracellular alkalinization paradoxically increases extracellular acidity, reinforcing the acidic TME.

Acid‐responsive nanomaterials represent a class of intelligent nanomaterials capable of recognizing and responding to the acidic conditions of the TME through structural or functional alterations. Based on their fundamental responsive mechanisms, these materials can be sub‐categorized into two primary types of chemical functionalities: i) Materials based on reversibly protonatable groups. These functionalities undergo a reversible change in their ionization state in response to pH shifts, leading to alterations in hydrophilicity, conformation, or membrane stability. This category includes materials composed of pH‐sensitive polymers (e.g., poly(acrylic acid) (PAA), chitosan), whose hydrophilic/hydrophobic properties or molecular conformations dynamically adjust;^[^ ^22^ ^]^ pH‐sensitive lipid‐based nanomaterials (e.g., pH‐sensitive liposomes containing ionizable lipids) that demonstrate enhanced membrane fusion or decomposition capabilities upon protonation under low‐pH conditions;^[^ ^23^ ^]^ and materials incorporating pH‐sensitive peptides or proteins whose molecular conformations undergo pH‐dependent structural transitions, exemplified by histidine‐rich nanocarriers.^[^ ^24^ ^]^ ii) Materials based on irreversibly acid‐responsive, hydrolysable groups. This category includes materials based on pH‐sensitive chemical bonds, wherein pH‐labile covalent linkages such as hydrazone, imine, acetal, ketal, oxime, as well as certain acid‐labile derivatives of amide and ester bonds (e.g., maleic acid monoamides, trityl esters) remain relatively stable under neutral or alkaline conditions but undergo hydrolysis or specific cleavage in the acidic TME, with the strategic incorporation of these acid‐unstable bonds in delivery system serving as a fundamental design approach for pH‐responsive nanomaterials to facilitate drug delivery to tumor tissues and subsequent release under acidic pH conditions;^[^ ^25^ ^]^ systems based on inorganic nanomaterials that are functionalized to be pH‐responsive, such as mesoporous silica NPs that loaded with therapeutic agents and capped with pH‐sensitive gates or those employing pH‐responsive metal–organic frameworks, which undergo dissolution or structural changes under acidic tumor conditions to enable controlled drug release.^[^ ^26^ ^]^ The rational design of these acid‐responsive nanomaterials leverages these distinct responsive mechanisms to promote tumor‐specific drug accumulation and ultimately enhance the efficacy of tumor immunotherapy.

### 2.2 Redox‐Induced Responsive Delivery

In neoplastic tissues, the equilibrium between reducing agents and oxidative molecules disrupted, leading to an abnormal accumulation of oxidative agents—particularly reactive oxygen species (ROS) and reactive nitrogen species. This is primarily driven by metabolic dysregulation and the overproduction of metabolites by immune cells. Tumor cells engage in high glucose uptake via aerobic glycolysis, generating substantial amounts of nicotinamide adenine dinucleotide phosphate (NADPH) and GSH. As a core antioxidant, GSH reaches significantly elevated intracellular concentrations.^[^ ^27^ ^]^ Concurrently, rapid tumor growth induces structurally abnormal and poorly perfused microvascular networks, impeding the clearance of oxidized GSH (GSSG) and other species, thereby exacerbating the reduced microenvironment.^[^ ^28^ ^]^ Furthermore, to counteract increased oxidative stress, the antioxidant systems is upregulated in tumor cells, resulting in elevated intracellular concentrations of reductive species sustain a highly reduced state.^[^ ^29^ ^]^ This unique redox homeostasis constitutes a critical driver of tumor drug resistance and metastasis.

The concurrent elevation of both oxidative and reductive species in the TME presents a seemingly paradoxical landscape for designing responsive materials. A critical consideration for leveraging this environment is the distinct chemical bonds and subcellular localization of these stimuli. The reductive potential is dominated by a high intracellular GSH concentration.^[^ ^17^ ^]^ This makes reduction‐sensitive linkages ideal for triggering drug release after cellular internalization and endosomal escape. The predominant strategy for imparting reduction sensitivity involves incorporating disulfide bonds. The reduction potential of these bonds aligns with the intracellular GSH/GSSG redox potential, enabling their reductive cleavage in the high‐GSH TME. This cleavage leads to material disintegration and subsequent release of active agents.^[^ ^30^ ^]^ Beyond disulfides, diselenide and nitroazo aryl ether group are also explored as GSH responsive materials. In contrast, oxidative stress, characterized by elevated ROS levels, is often associated with mitochondria, the endoplasmic reticulum, and particularly the extracellular matrix of aggressive tumors.^[^ ^28b^ ^]^ Oxidation‐sensitive strategies are mainly use thioketals, thioether bond, or aryl boronate esters. Oxidation‐responsive systems are relevant for targeting the TME or for designing organelle‐specific delivery systems due to their potential sensitivity to the accumulated ROS.^[^ ^31^ ^]^ The choice between reduction‐responsive and oxidation‐responsive designs is not mutually exclusive but should be guided by the pharmacological target of the drug and the structure of the system. For example, for drugs acting on cytoplasmic targets, GSH‐responsive systems are paramount. Advanced systems are now being developed to sequentially or synergistically respond to both stimuli, enhancing both specificity and efficacy.^[^ ^32^ ^]^

### 2.3 Nanomaterials Designed for Enzyme‐Responsive Delivery

Enzymes play indispensable roles in nearly all biological processes and function as highly effective triggers for targeted drug activation, particularly within TME.^[^ ^33^ ^]^ The pathological overabundance of diverse enzyme classes, including matrix metalloproteinases (MMPs), hyaluronidase, cathepsin B, IDO1, esterase, carbonic anhydrases, fibroblast activation protein‐α, and γ‐glutamyl transpeptidases, within malignant tissues and their associated stromal compartments has driven the innovation of enzyme‐activated therapeutic modalities.^[^ ^33^, ^34^ ^]^ For example, the expression level of hyaluronidase in well‐differentiated bladder cancer tissues is ≈8‐fold higher than that in normal bladder tissues.^[^ ^35^ ^]^

Researchers have engineered novel enzyme‐responsive nanomaterials by embedding enzyme‐specific cleavage motifs within their structural framework. These strategically incorporated peptide sequences undergo targeted hydrolysis upon contact with the corresponding enzyme, generating distinct material responses, including complete disintegration of the nanoparticulate system,^[^ ^36^ ^]^ selective exposure of embedded targeting elements through chain scission,^[^ ^37^ ^]^ or controlled aggregation of NP components.^[^ ^38^ ^]^ The specific response is governed by the spatial configuration of the cleavage sites within the polymeric matrix, enabling precise modulation of material behavior. By capitalizing on enzyme‐specific cleavage domains strategically embedded within biomaterial constructs, these systems enable regulated delivery of diagnostic contrast agents, prognostic indicators, therapeutic efficacy monitors, and immunomodulatory compounds.^[^ ^34^, ^39^ ^]^ Simultaneously, this molecular engineering approach permits the deconstruction of therapeutic assemblies into diminished molecular entities, thereby substantially augmenting their ability to traverse the obstructive extracellular matrix characteristic of solid tumors.^[^ ^40^ ^]^

### 2.4 Hypoxia Environment and Hypoxia‐Responsive Nanomaterials

Carcinogens and an acidic TME induce tumor vasculature dysfunction, damaging endothelial cells and disrupting oxygen delivery. The heightened metabolic demand of cancer cells intensifies hypoxia when consumption surpasses supply. Aberrant angiogenesis generates malformed, non‐functional vessels, compounding oxygen deprivation even with elevated erythropoietin/angiogenic factors.^[^ ^41^ ^]^ Rapid tumor expansion establishes chronic hypoxia. Stromal remodeling (e.g., fibroblast activation, fibrin deposition) compresses vasculature, impairing perfusion and fostering thrombosis.^[^ ^42^ ^]^ Magnesium inhibits angiogenesis under hypoxic conditions, further accelerating TME hypoxia.^[^ ^43^ ^]^ Numerous nanomaterials are designed to directly respond to hypoxia, while cargo release mechanisms in other systems rely on detecting hypoxia‐induced metabolites. The predominant hypoxia‐sensitive constituents, including quinone, nitroaromatic, and azobenzene moieties, form the structural basis of most nanomedicines engineered for hypoxia responsiveness.^[^ ^44^ ^]^ In addition, bacteria‐mediated hypoxia‐specific NPs display favorable prospects as hypoxia‐specific therapeutics that enrich the efficacy of immunotherapy.^[^ ^45^ ^]^

### 2.5 High‐Level of ATP and ATP‐Responsive Nanomaterials

Under stressors including autophagy and oxygen deprivation, cellular ATP secretion occurs.^[^ ^46^ ^]^ This extracellular nucleotide stimulates angiogenesis, a process potentially initiated during hypoxic conditions to foster vasculogenesis. Malignant cell lysis (via autophagy or immune‐mediated killing) liberates ATP through passive diffusion, driven by steep concentration gradients between intracellular and extracellular compartments. Alternatively, active export mechanisms like vesicular export and transporter‐mediated efflux facilitate ATP release. Crucially, human umbilical vein endothelial cell studies demonstrated hypoxia enhanced exocytotic ATP export.^[^ ^47^ ^]^ Both direct and indirect quantification methods consistently measure tumor microenvironmental ATP at hundreds of micromolar, contrasting sharply with nanomolar levels in healthy tissues.^[^ ^48^ ^]^ Harnessing this biochemical stimulus as an activation signal or metabolic fuel holds significant promise for advancing immunotherapeutic nanodevices.

ATP‐responsive nanomaterials leverage high‐affinity interactions between the polyphosphate backbone of ATP and specific functional units, including aptamers and cationic groups, for targeted design. Aptamer‐based systems exploit conformational changes upon nucleotide binding to trigger drug release with high specificity.^[^ ^49^ ^]^ Phenylboronic acid (PBA)‐containing polymers form reversible diol complexes with therapeutic agents, and elevated nucleotide concentrations induce competitive displacement of oligonucleotides for stimulus‐responsive delivery.^[^ ^50^ ^]^ Crucially, metal‐ATP coordination bonds enable dynamic cyclic responsiveness. Metal‐coordination architectures employ zinc‐functionalized mesoporous silica with polypeptide gatekeepers that seal nanopores until ATP‐triggered displacement of chelated chains enables controlled cargo liberation.^[^ ^51^ ^]^ These approaches establish programmable spatiotemporal control for oncological nanomedicine. Notwithstanding the immunosuppressive backdrop of the TME, dominated by the adenosine pathway,^[^ ^52^ ^]^ the therapeutic rationale for ATP‐responsive nanomaterials lies in exploiting a critical spatiotemporal window. Extracellular ATP serves as a transient ‘danger signal’ released during immunogenic cell death (ICD) or therapy‐induced killing.^[^ ^53^ ^]^ These systems are designed to trigger drug release in response to this initial ATP burst, thereby amplifying immunostimulation before nucleosidases hydrolyze ATP into immunosuppressive adenosine.^[^ ^46b^ ^]^ This strategy aims to leverage a proximal immunogenic cue to disrupt the distal immunosuppressive network. Despite their potential, clinical translation is challenged by heterogeneous ATP distribution causing inconsistent drug release, and off‐target effects in non‐malignant inflamed tissues. Future designs require spatiotemporally controlled sensing architectures. Overcoming these hurdles requires the rational engineering of spatially and temporally controlled ATP‐sensing architectures to precisely orchestrate immunomodulatory actions within the tumor niche.

### 2.6 Multiple‐Responsive Nanomaterials

Multi‐stimuli‐responsive nanomaterials integrate diverse functional units like pH‐sensitive moieties, GSH‐responsive polymers, and reduction‐cleavable linkers into single nanocarriers via covalent conjugation or physical encapsulation. This multifunctional integration enables spatiotemporal control over TME through orthogonal activation mechanisms, significantly enhancing drug delivery efficiency, therapeutic precision, and off‐target effect mitigation. Dual‐responsive systems are well‐established in cancer immunotherapy, for example, pH/GSH,^[^ ^54^ ^]^ pH/enzyme,^[^ ^55^ ^]^ pH/ROS,^[^ ^56^ ^]^ GSH/ROS,^[^ ^57^ ^]^ GSH/enzyme^[^ ^58^ ^]^ dual‐responsive nanomaterials. Recent advances further yield triple‐responsive platforms (e.g., pH/enzyme/GSH‐activated delivery systems)^[^ ^59^ ^]^ for immunomodulation.

Among the multiple‐responsive systems, pH/ROS and pH/enzyme dual‐responsive platforms stand out as particularly compelling. The pH/ROS combination is highly synergistic, as mild acidity can act as a priming signal that sensitizes the carrier to subsequent, more specific ROS‐triggered drug release, thereby creating a sophisticated two‐step activation logic that maximizes specificity. Similarly, pH/enzyme systems exploit the spatial hierarchy of TME stimuli, where acidity marks the general tumor region and enzyme overexpression like MMPs pinpoints the most aggressive, invasive fronts. These combinations effectively function as biological ‘AND’ gates, requiring the coincidence of two signals to actuate release, which dramatically reduces off‐target effects in healthy tissues. While triple‐responsive systems represent a tour de force in materials engineering, their increased complexity often poses formidable challenges for reproducible manufacturing and pharmacokinetic control, potentially hindering clinical translation. Therefore, the future advancement of novel TME‐responsive nanomedicines may lie not in the maximal number of triggers, but in the intelligent selection of minimally sufficient, synergistic stimulus pairs that best exploit the pathological logic of the TME. **Table** **1** lists the commonly used responsive biochemical linkers.

**Table 1:** Summary of commonly used responsive biochemical linkers.

Type	Linkage	Structure	Refs.
pH responsive	Acetal and ketal bond	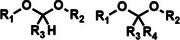	[60]
	Imine	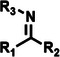	[61]
	Hydrazone	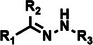	[62]
	Orthoester linkage	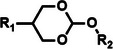	[63]
	Cis‐aconityl group	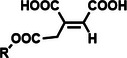	[64]
ROS responsive	Thioacetal and thioketal linkage	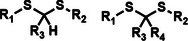	[65]
	Thioether bond	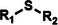	[66]
	Boronic ester linkage	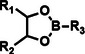	[67]
	Selenium ether and tellurium ether bond	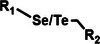	[68]
	Diselenide	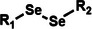	[69]
GSH responsive	Disulfide linker	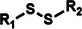	[70]
	Diselenide	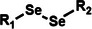	[71]
	Nitroazo aryl ether group	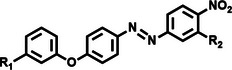	[72]
Enzyme responsive	Enzyme‐cleavable peptide linkers, e.g. GFLG (cathepsin B), PLGLAG (MMP), DEVD (caspase 3)	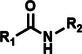	[73]
	Specific ester‐based motifs, e.g. phenyl esters, α‐cyano esters (carboxylesterases)	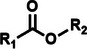	[74]
	Glycosidic bond	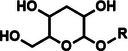	[75]
	Phosphate ester bond	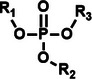	[76]
Hypoxia responsive	Azo linkage	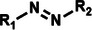	[77]
	Quinone moiety	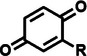	[78]
	Nitro group	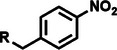	[79]
ATP responsive	Adaptor‐ligand recognition structure	ATP‐ nucleic acid aptamers	[49, 80]
	Metal coordination bond	X‐Zn^2+^ complex	[51, 81]

## 3 Mechanisms of TME‐Responsive Nanomaterials Potentiating Antitumor Immunity

**Figure 1:**
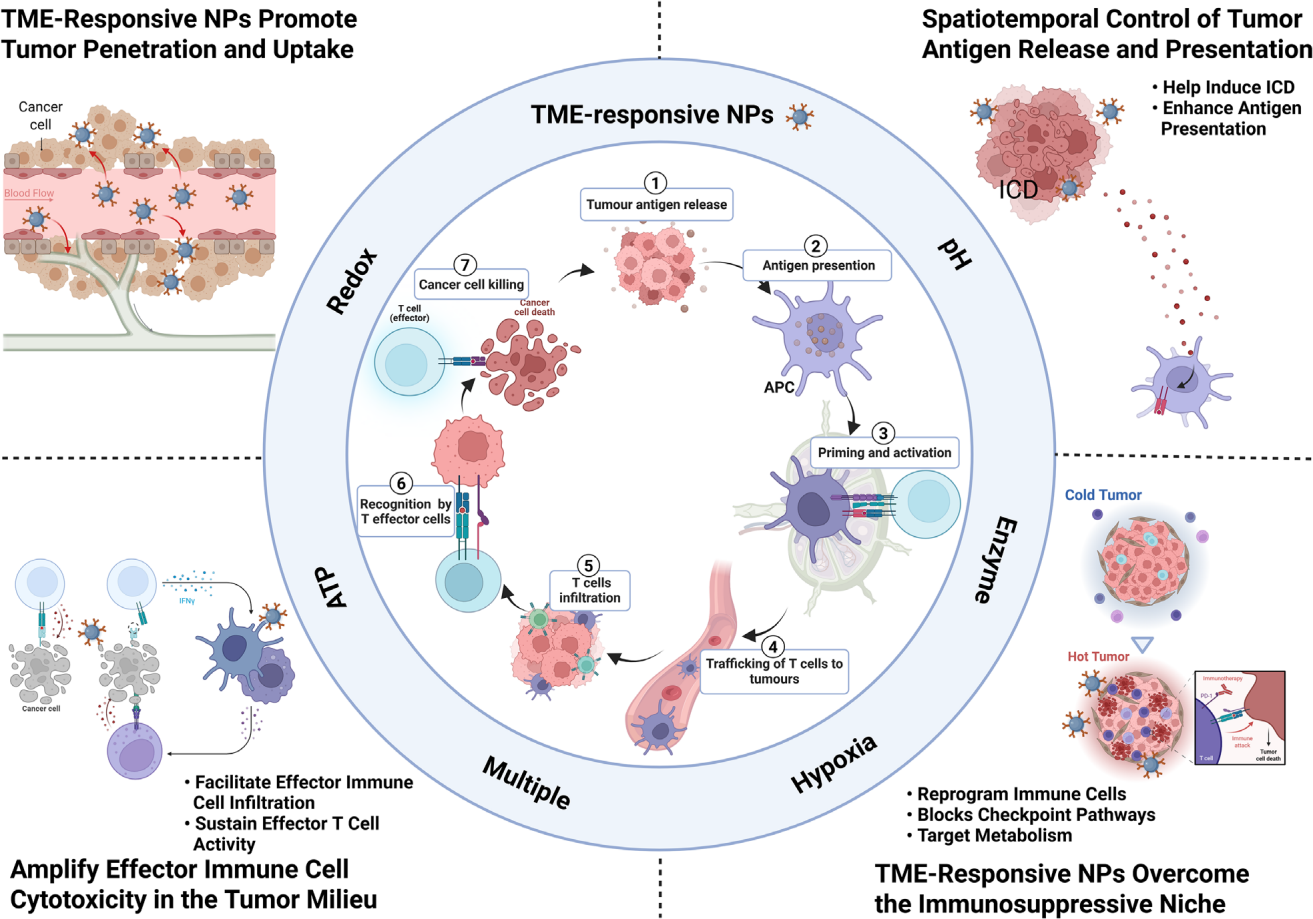
Summary of strategies for TME‐responsive nanomaterials to augment cancer immunotherapy. Created with BioRender.com released under a Creative Commons Attribution‐NonCommercial‐NoDerivs 4.0 International license.

Cancer immunotherapy fundamentally mobilizes the body's intrinsic anti‐tumor immunity via the dynamically regulated cancer‐immunity cycle. This cycle initiates with the priming phase, where dendritic cells (DCs) and other antigen‐presenting cells (APCs) capture, process, and present tumor antigens to T cells. It progresses to the activation phase, during which co‐stimulatory signals trigger T cell activation, clonal expansion, and differentiation. The cycle culminates in the effector phase as CTLs infiltrate tumor lesions to execute tumor‐specific killing.^[^ ^82^ ^]^ However, immunosuppressive TME frequently disrupt this cycle, limiting efficacy. TME‐responsive nanomaterials overcome this barrier through intelligent adaptation to local pathological conditions. These systems enhance antigen delivery and cross‐presentation during priming, reprogram immunosuppressive microenvironments to potentiate T cell activation, and sustain CTL infiltration with functional persistence in the effector phase, thereby systemically augmenting the efficiency and controllability of cancer immunotherapy (**Figure** **1**).

TME‐responsive nanomaterials enhance the efficacy of immunotherapy through various steps of the immune cycle. Certainly, while a valuable conceptual model, the cancer‐immunity cycle is in reality a deeply interconnected network, not a linear sequence. Dysfunction at any point propagates system‐wide, and therapeutic interventions inevitably have pleiotropic effects across multiple stages. For instance, an immunosuppressive metabolic microenvironment not only directly inhibits T‐cell function but also alters the differentiation and polarization of APCs, thereby impairing antigen presentation and T‐cell priming. Conversely, successful immune cell reprogramming will fundamentally reshape the metabolic and cytokine landscape of the TME, simultaneously impacting antigen presentation, T‐cell infiltration, and effector function. Therefore, the TME‐responsive nanotherapeutic strategies discussed in the following sections are often deliberately target these key interdependencies to catalyze a self‐reinforcing cycle of immune activation, moving beyond single‐step targeting.

### 3.1 TME‐Responsive Nanomaterials Promote Tumor Penetration and Uptake

Effective tumor penetration and cellular uptake are critical prerequisites for nanomedicines to exert optimal antitumor immunotherapeutic effects. The pathological microenvironment of solid tumors, characterized by the dense extracellular matrix (ECM), elevated interstitial fluid pressure (IFP), and aberrant vasculature, severely impedes deep intratumoral penetration and efficient cellular internalization of nanotherapeutics, thereby limiting their immunotherapeutic efficacy.^[^ ^83^ ^]^ Engineering nanomaterials to undergo TME‐responsive dynamic structural transformations and controlled drug release can overcome these barriers, enhancing deep‐tissue penetration and cellular uptake.

**Figure 2:**
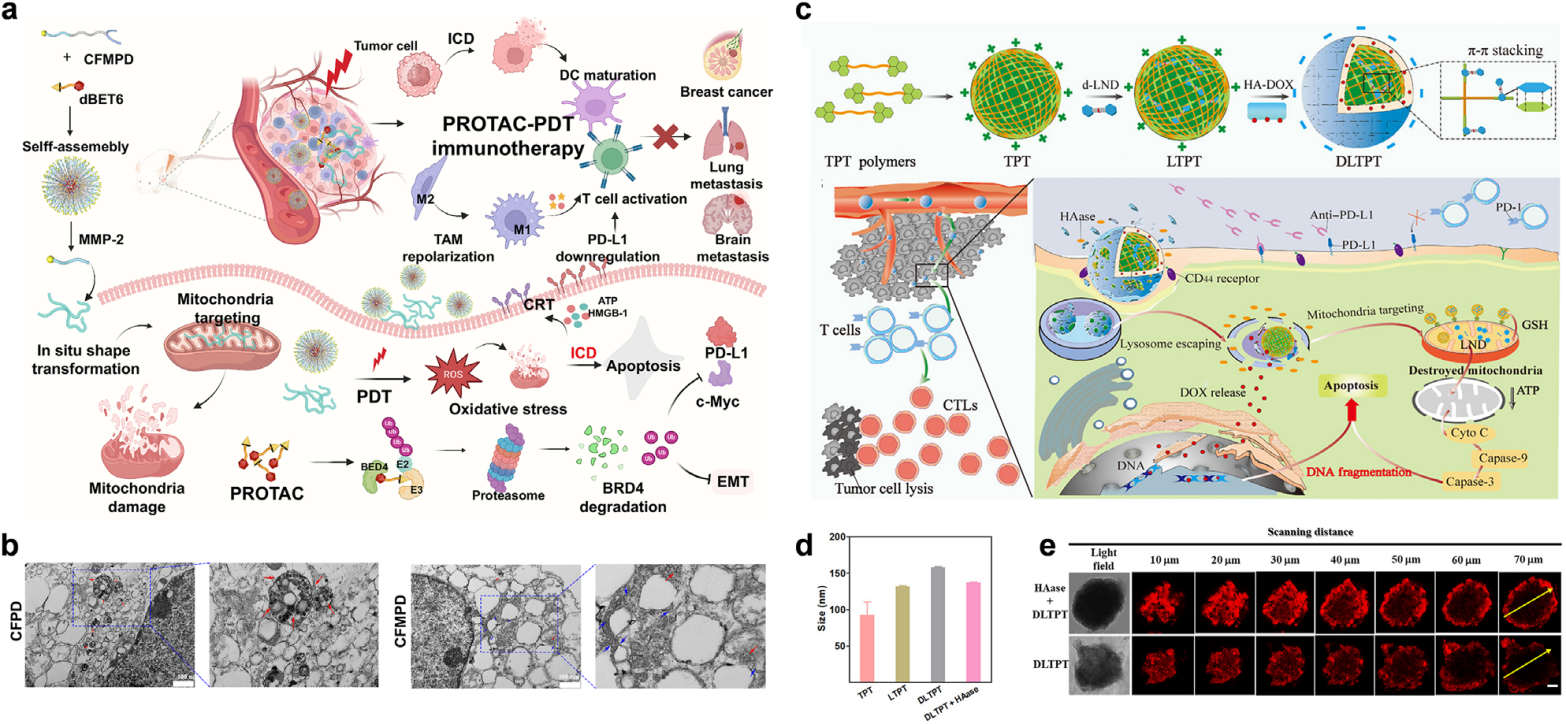
TME‐ responsive nanomaterials promote tumor penetration and uptake. a) Schematic illustration of the composition of dBET6@CFMPD and its therapeutic effect on primary breast cancer and brain metastases. b) The TME images and enlarged images of cells treated with CFPD and CFMPD. Red arrows and blue arrows indicate spherical NPs and nanofibers, respectively (scale bar = 500 nm). Reproduced with permission.^[^ ^89^ ^]^ Copyright 2025, Springer Nature. c) Schematic diagram of dual‐drug chemo‐ and immune‐combinational therapy mechanism. d) Particle sizes of TPT, LTPT, DLTPT, and HAase + DLTPT. e) CLSM images of DLTPT and HAase + DLTPT (incubation with HAase for 4 h) incubated with 4T1 multicellular tumor spheroids for 6 h (scale bar, 100 µm). Reproduced with permission.^[^ ^92^ ^]^ Copyright 2021, AAAS.

Strategies to improve intratumoral permeation and retention primarily leverage TME‐triggered mechanisms, including size and charge switching of nanocarriers,^[^ ^84^ ^]^ and on‐demand release of ECM‐remodeling agents^[^ ^85^ ^]^ or vasoactive regulators^[^ ^86^ ^]^ to normalize the tumor stroma and reduce IFP. Additionally, intratumoral infiltration can be enhanced by promoting the transcytosis of tumor cells.^[^ ^87^ ^]^ These approaches collectively enhance the permeation and retention of nanodrugs within tumor tissues. In the previous work, we designed a series of TME‐responsive shape‐variable nanomedicines to promote the penetration and retention of tumors.^[^ ^88^ ^]^ Building on prior work, we engineered an MMP‐2‐responsive, shape‐transformable, and mitochondria‐targeting nanoplatform (dBET6@CFMPD) leveraging the high MMP‐2 protease expression in TME. Characterization confirmed dBET6@CFMPD as monodisperse, stable spherical NPs. Upon reaching tumor sites, these sub‐100 nm NPs distribute and penetrate deep into tumor tissues. When exposed to elevated MMP‐2 levels in the TME, the NPs undergo a programmed morphological transition from spheres to nanofibers, concomitant with controlled release of the PROTAC molecular degrader dBET6. This transformation significantly enhances intratumoral distribution and retention of dBET6, and accumulation of the photosensitizer component. Consequently, the platform amplifies photodynamic therapy (PDT) efficacy, promoting ICD of tumor cells and ultimately triggering potent antitumor immunity (**Figure** **2**a,b).^[^ ^89^ ^]^ For augmenting cellular internalization, key methods involve stimulus‐responsive exposure of targeting ligands^[^ ^90^ ^]^ and pH‐mediated charge reversal,^[^ ^91^ ^]^ both significantly boosting endocytic efficiency in tumor cells. In another previous work, we engineered dual‐drug‐loaded core–shell NPs (DLTPT) with hierarchical targeting capabilities. DLTPT exhibits prolonged blood circulation and efficient tumor accumulation due to its tumor‐homing properties and anionic hyaluronic acid coating. Subsequent degradation of the HA‐doxorubicin (HA‐DOX) shell by extracellular hyaluronidase triggers particle size reduction and surface charge reversal from negative to positive, significantly enhancing tumor penetration and cellular internalization to promote therapeutic efficacy (Figure 2c–e).^[^ ^92^ ^]^ By synergistically enhancing intratumoral accumulation and cellular uptake, these strategies establish a targeted delivery foundation essential for subsequent immunotherapeutic activation.

### 3.2 Initiation Phase: Spatiotemporal Control of Tumor Antigen Release and Presentation

The initiation phase of cancer immunotherapy critically relies on robust tumor antigen release and its efficient delivery to lymphoid organs for presentation, processes that directly govern the activation efficacy of adaptive immune responses.^[^ ^93^ ^]^ Conventional therapies often fail to effectively prime immunity due to insufficient antigen exposure and functional impairment of APCs.^[^ ^94^ ^]^ By engineering TME‐responsive nanomaterials, the release of antigens can be regulated spatiotemporally and antigen presentation efficiency enhanced, thereby overcoming fundamental limitations of current immunotherapy approaches. TME‐responsive nanomaterials can be engineered enable spatiotemporally controlled release of tumor antigens within the TME, and facilitate the drainage and targeted delivery of these antigens to lymph nodes for presentation. This is often achieved by designing carriers that are optimally sized for lymphatic uptake or that enhance the capture and migration of antigen‐loaded dendritic cells. By enhancing both antigen availability and its delivery to the sites of T‐cell priming, these nanomaterials can fundamentally improve the initiation of antitumor immunity.

#### 3.2.1 TME‐Responsive Nanomaterials Induce ICD

**Figure 3:**
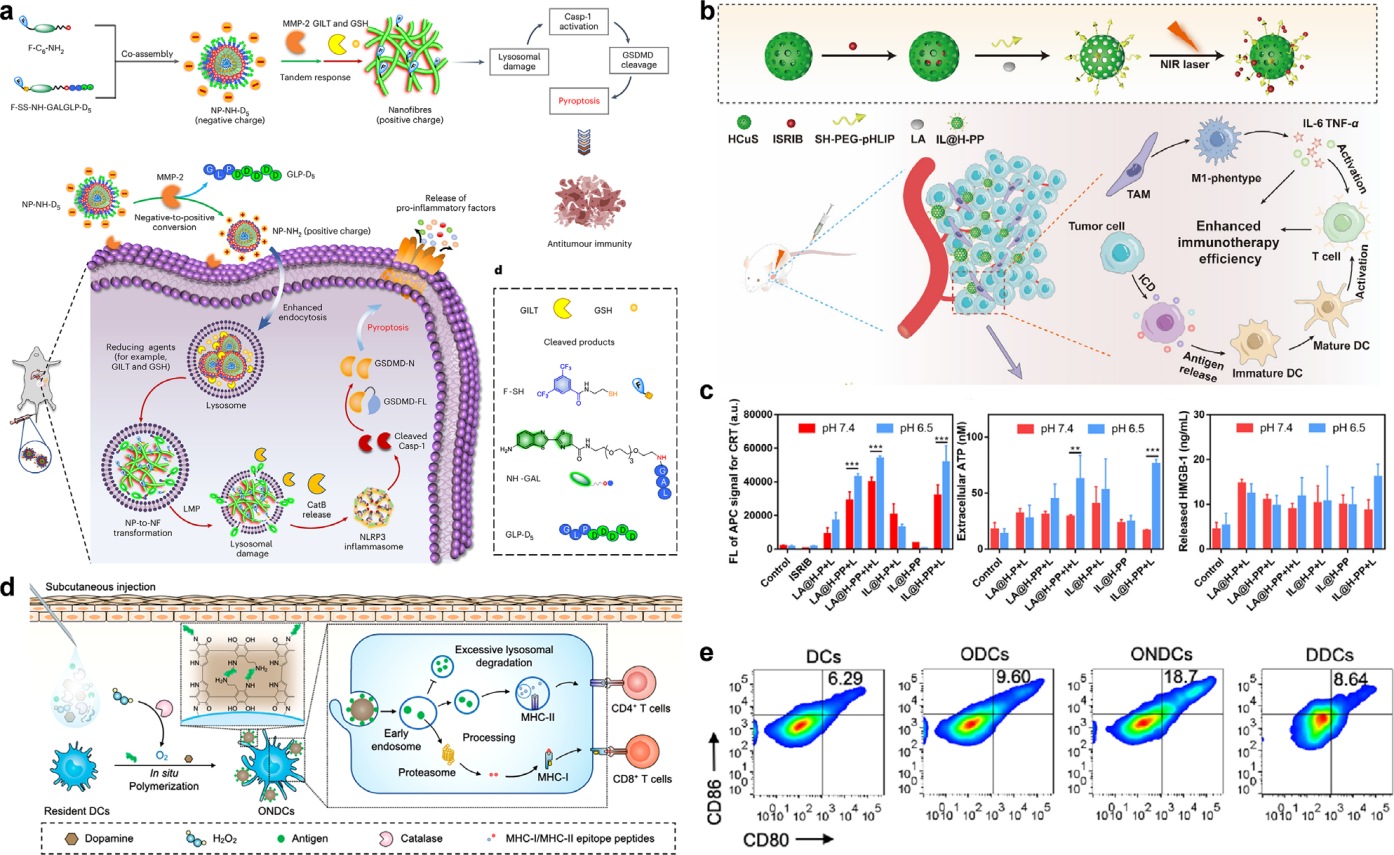
The examples of TME‐responsive nanomaterials that achieve spatiotemporal control of antigen release and presentation. a) Preparation of NP‐NH‐D5 via molecular co‐assembly of F‐C6‐NH_2_ and F‐SS‐NH‐GALGLP‐D5, and proposed transformation of spherical NPs into nanofibers to induce GSDMD‐mediated pyroptosis in tandem response to MMP‐2 and reducing agents. Blow is the schematic illustration of NP‐NH‐D5 to selectively deliver F‐C6‐NH_2_ to tumor cells for in vivo cancer immunotherapy. The release of pro‐inflammatory factors enhancing the ICD effect. This process creates immunogenic ‘hot’ tumours for effective antitumour immunity. Reproduced with permission.^[^ ^96^ ^]^ Copyright 2025, Springer Nature. b) Scheme illustration of the composition of IL@H‐PP and its therapeutic effect on primary breast cancer and brain metastases. c) CRT translocation on 4T1 cells was detected by flow cytometry analysis. Reproduced with permission.^[^ ^95c^ ^]^ Copyright 2022, Elsevier. d) In vivo deposition of antigens on the surface of subcutaneous DCs is achieved by in situ dopamine polymerization, promoting antigen presentation by decreasing lysosome‐associated antigen degradation, and elucidating antigen‐specific protective immune responses. e) Results of flow cytometry contour plots of percentage of CD80^+^CD86^+^ BMDCs after incubation of NPs in d. Reproduced with permission.^[^ ^103^ ^]^ Copyright 2023, American Chemical Society.

TME‐responsive nanomaterials enable spatiotemporally controlled drug release triggered by TME‐specific signals, inducing ICD in tumor cells to establish the foundation for adaptive immune activation. Most research focuses on stimuli‐responsive delivery systems that enhance ICD induction agents through mechanisms such as endoplasmic reticulum (ER) stress and mitochondrial damage. These promote calreticulin (CRT) exposure on plasma membranes and release of damage‐associated molecular patterns (DAMPs), generating autologous vaccine‐like signals.^[^ ^95^ ^]^ These DAMPs act in concert with tumor antigens to activate DCs, thus inducing killer T cells that eliminate tumor cells. Ye's group developed tumor‐specific NPs (NP‐NH‐D5) that undergo cascade reactions to extracellular matrix metallopeptidase‐2 and intracellular reductants within tumor cells. This triggers charge reversal (negative to positive) and morphological transformation (NPs to nanofibers) in lysosomes. The resulting non‐peptidic nanofibers effectively disrupt lysosomal membranes, activating gasdermin‐D‐mediated pyroptosis. This induces potent ICD and substantially enhances the efficacy of combinatorial antibody immunotherapy (**Figure** **3**a).^[^ ^96^ ^]^ By leveraging TME‐responsive ICD induction, such nanomaterials not only provide abundant tumor antigen reservoirs for immune recognition but also minimize systemic toxicity through spatiotemporal drug control. This establishes an efficient and safe antigen source for cancer immunotherapy. Concurrently, smart nanocarriers with responsive properties can be leveraged for the delivery of exogenous tumor antigens^[^ ^97^ ^]^ and adjuvants,^[^ ^98^ ^]^ enabling on‐demand antigen replenishment to enhance immunotherapeutic efficacy.

#### 3.2.2 TME‐Responsive Nanomaterials Enhance Antigen Presentation

Following antigen release, efficient capture, processing, and presentation by APCs, primarily DCs, are essential for T‐cell activation. However, the TME exhibits pronounced immunosuppressive properties and physiological barriers that constrain immunotherapeutic efficacy. To overcome these challenges, TME‐responsive nanomaterials incorporate environmentally sensitive motifs enabling precise spatiotemporal release of antigens and adjuvants within tumor tissue and promote their delivery to lymphoid organs for presentation. Through targeted recruitment of APCs to tumor sites,^[^ ^99^ ^]^ augmented antigen internalization and processing,^[^ ^100^ ^]^ enhanced lymphatic drainage to facilitate antigen delivery to lymph nodes,^[^ ^101^ ^]^ and coordinated activation of APC maturation,^[^ ^102^ ^]^ TME‐responsive nanomaterials enhance DC functions and potentiate DC antigen uptake and cross‐presentation efficiency. Liu et al. developed an in situ polymerization‐mediated antigen presentation (IPAP) strategy through dopamine polymerization on APC surfaces. This approach enables codeposition of tumor antigens with dopamine monomers, generating antigen‐loaded NPs that adhere efficiently to DCs. Consequently, DCs exhibit enhanced antigen uptake and improved cross‐presentation to T lymphocytes (Figure 3d,e).^[^ ^103^ ^]^ Stimuli‐responsive nanocarriers can be exploited to deliver immunomodulators for controlled release within the TME, directly triggering costimulatory molecule expression on DCs to drive their maturation into APCs. This approach concurrently enhances antigen cross‐presentation, eliciting robust T cell responses. Building upon the acidic tumor niche, researchers engineered a pH‐responsive guanidyl‐rich stimulator of interferon genes (STING) nanoagonist (nPGSA) that efficiently delivers cGAMP to the ER. This system enables precise STING pathway activation, catalyzing DC maturation to promote tumor antigen cross‐presentation to CD8⁺ T cells and amplify antitumor immunity.^[^ ^104^ ^]^

### 3.3 Activation Phase: Overcoming the Immunosuppressive Niche

Eliciting tumor antigen‐specific immune responses represents a critical objective of successful immunotherapy. Ideally, the generated antigen‐specific T cells should not only effectively eliminate primary tumor cells but also eradicate tumor cells within distant metastases. The TME, a unique niche enabling tumor cell survival and evolution, exhibits high heterogeneity and complex cellular composition.^[^ ^14b^ ^]^ This milieu not only facilitates tumor growth, invasion, and metastasis but also actively establishes an immunosuppressive state through diverse mechanisms, thereby driving resistance to immunotherapy.^[^ ^105^ ^]^ TME‐responsive NPs overcome this challenge by precisely recognizing TME‐specific pathophysiological signals to achieve targeted drug release or functional activation within the tumor. This effectively reverses immunosuppression and enhances immunotherapeutic efficacy. Strategically designed to counteract the immunosuppressive TME, these NPs primarily leverage: reprogramming immunosuppressive cells, blocking immune checkpoint pathways, neutralizing immunosuppressive cytokines, and modulating dysregulated tumor metabolism. These approaches collectively aim to reshape the immunological landscape of the TME and promote the activation of immune cells to improve immunotherapy response rates.

#### 3.3.1 TME‐Responsive Nanomaterials Reprogram Immunosuppressive Cells

**Figure 4:**
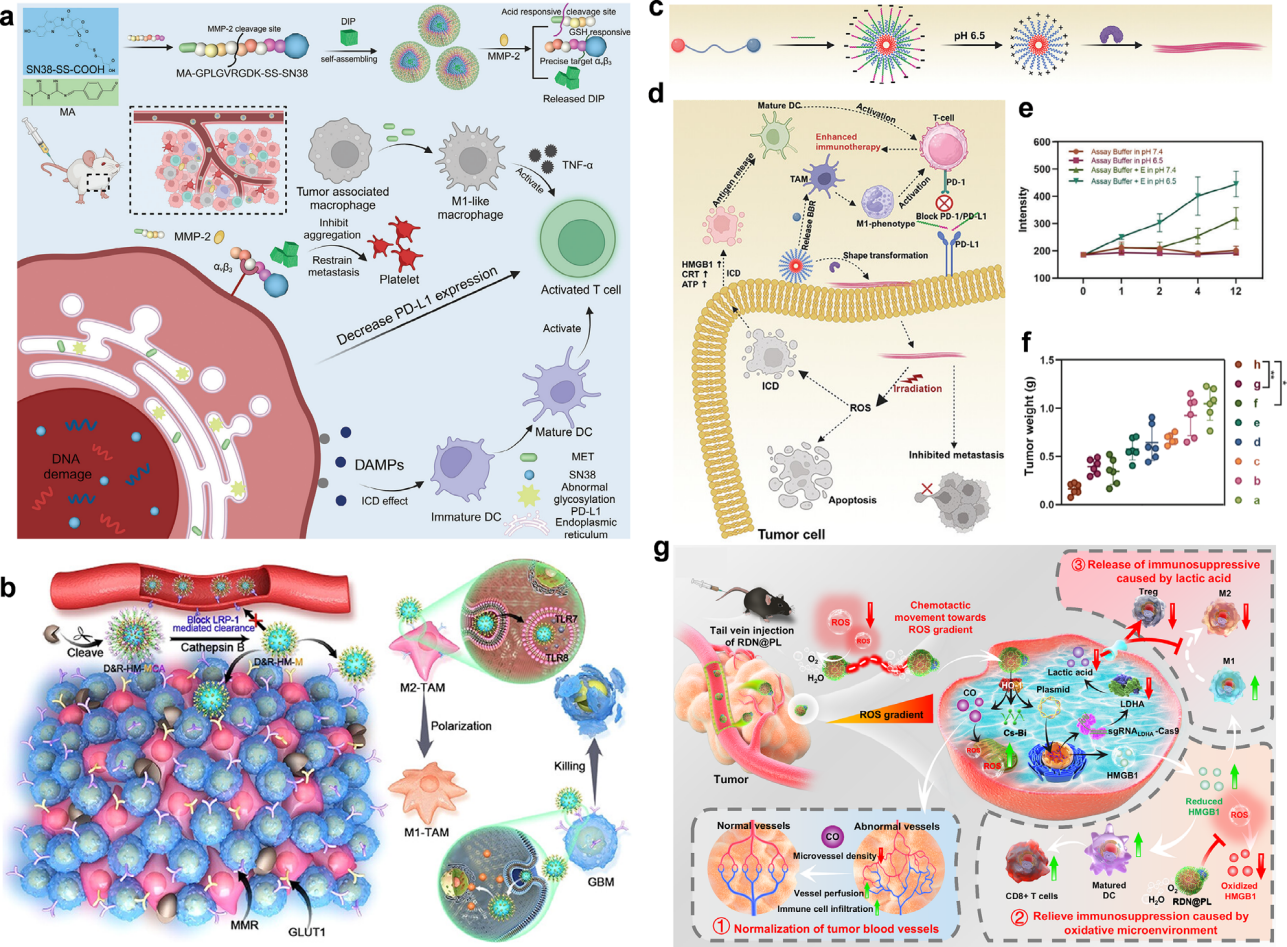
The examples of TME‐responsive nanomaterials overcoming the immunosuppressive niche. a) Diagram depicting the design of the spatial‐specific multi‐responsive carrier‐free self‐assembled nanodrug delivery system for synergistic breast cancer therapy. Reproduced with permission.^[^ ^108^ ^]^ Copyright 2024, Wiley. b) The targeting mechanism, and pharmacological action of D&R‐HM‐MCA. Reproduced with permission.^[^ ^109^ ^]^ Copyright 2024, American Chemical Society. c) Scheme illustration of the composition of DHP@BPP, as well as d) the mechanism of the chemo‐photodynamic‐immunotherapy. e) Intensity changes of DHP@BPP in different conditions. f) Tumor weights of 4T1‐bearing mice in each group during treatment. Reproduced with permission.^[^ ^115^ ^]^ Copyright, 2024, Wiley. g) Schematic illustration of the synergistic solid tumor therapy that extracellular regulation and intracellular metabolic disruption by RDN@PL. Reproduced with permission.^[^ ^118^ ^]^ Copyright 2025, Springer Nature.

The abnormal vasculature and uncontrolled proliferation of cancer cells within the TME create severe oxygen deprivation. This hypoxic condition fosters the recruitment and survival of immunosuppressive populations, including regulatory T cells (Tregs), myeloid‐derived suppressor cells (MDSCs), and tumor‐associated macrophages (TAMs).^[^ ^106^ ^]^ Among TAMs, the M1 phenotype exhibits pro‐inflammatory and antitumor activities, while the M2 subtype promotes immune tolerance and tumor progression. These suppressive cells further exacerbate immunosuppression by releasing cytokines such as TGF‐β, VEGF, and interleukin (IL)‐10.^[^ ^107^ ^]^ To improve the effectiveness of cancer vaccination strategies, NP‐based approaches can be employed to either selectively eliminate these suppressive cells or reprogram them into immune‐stimulatory phenotypes. To maximize therapeutic efficacy, our team developed a multi‐responsive, carrier‐free self‐assembled nanoplatform enabling spatiotemporal precision targeting for compartmentalized drug release. This system reverses breast cancer immunosuppression by repolarizing M2‐like macrophages within the TME. The NP was engineered through Schiff base conjugation of metformin (MET) and 7‐ethyl‐10‐hydroxycamptothecin (SN38) to an MMP‐2‐cleavable peptide (GPLGVRGDK) via terephthalaldehyde bridges, with subsequent co‐assembly incorporating hydrophobic dipyridamole (DIP). Upon encountering tumor‐overexpressed MMP‐2, NPs undergo proteolytic cleavage yielding three bioactive components: DIP binds circulating platelets to mitigate metastasis; the pH‐responsive MA‐GPLG moiety releases MET in acidic niches to downregulate PD‐L1 and repolarize M2 macrophages; while the αvβ3 integrin‐targeting VRGDK‐SS‐SN38 conjugate undergoes GSH‐triggered dissociation in tumor cells, unleashing SN38 for precise cytotoxicity. This multi‐stage activation cascade achieves spatially controlled drug deployment, wherein the combinatorial action of SN38, MET, and DIP synergistically overcomes immunosuppressive barriers, significantly enhancing therapeutic response rates (**Figure** **4**a).^[^ ^108^ ^]^ In a parallel study, we engineered a cathepsin B‐responsive cascade‐targeting nanoplatform (D&R‐HM‐MCA) for dual modulation of TAMs and glioblastoma multiforme (GBM). This system leverages proteolytic cleavage by tumor‐overexpressed cathepsin B to expose surface‐conjugated p‐aminophenyl‐α‐D‐mannopyranoside (MAN), enabling specific recognition of macrophage mannose receptors (MMR) on TAMs. This nanosystem simultaneously achieves chemotherapeutic eradication of GBM cells and potent repolarization of TAMs from immunosuppressive M2 to immunostimulatory M1 phenotypes, thereby remodeling the tumor immune microenvironment and enhancing anti‐tumor immunity against GBM (Figure 4b).^[^ ^109^ ^]^ Collectively, TME‐responsive nanomaterials can be engineered to systemically administer therapeutics to silence key immunosuppressive populations within the TME and potentiate the efficacy of tumor immunotherapy.

Effective tumor immunotherapy requires not only the activation of antitumor effector cells but also cytokines to promote T‐cell development.^[^ ^110^ ^]^ Immunosuppressive cells actively maintain immune homeostasis by secreting inhibitory molecules that suppress effector immune cell functions.^[^ ^111^ ^]^ Consequently, targeted cytokine modulation through microenvironment‐responsive nanosystems can reprogram immunosuppressive cells to potentiate antitumor immunity. Building on this concept, researchers engineered hematopoietic stem cells using a lentiviral vector platform for TME‐mediated gene delivery. This approach induced systemic release of interferon α (IFN‐α) or IL‐12, which effectively reprogrammed the glioblastoma TME into an immunostimulatory state, thereby suppressing tumor growth and improving survival rates.^[^ ^112^ ^]^ Furthermore, tumor‐secreted IL‐12 amplified the proliferation, functional priming, and cytolytic capacity of neoantigen‐reactive T lymphocytes, culminating in robust tumor regression.^[^ ^110b^ ^]^

#### 3.3.2 TME‐Responsive Nanomaterials Block Immunosuppressive Checkpoint Pathways

T cell proliferation and activation are precisely controlled through a complex interplay of stimulatory and inhibitory molecular signals. Key inhibitory signals are mediated by several immune checkpoint proteins, such as PD‐1, CTLA‐4, B and T lymphocyte attenuator (BTLA), IDO1, and CD47. Malignant cells frequently exploit these regulatory pathways within neoplastic tissues to circumvent host immune surveillance. Recent evidence suggests that synergistic targeting of multiple immune checkpoint pathways enhances therapeutic outcomes in cancer immunotherapy.^[^ ^113^ ^]^ Nevertheless, current limitations of checkpoint inhibition strategies include rapid systemic clearance, poor tumor localization, and potential adverse effects.^[^ ^114^ ^]^ Responsive local delivery of immune checkpoint inhibitory agents in combination with other drugs is a potential method for improving tumor immune response. We developed a dual‐responsive shape‐converting charge‐reversal integrated nanoplatform (DHP@BPP) for the co‐delivery of the photosensitizer pyropheophorbide‐α (Ppa), an anti‐programmed death‐ligand 1 peptide (dPPA), and a TAM‐modulating agent. This nanosystem responds to the TME by responsively exposing peptides to reduce PD‐L1 expression, thereby remodeling the immunosuppressive TME and synergistically potentiating ICD (Figure 4c–f).^[^ ^115^ ^]^ Meanwhile, Tang et al. engineered a pH‐sensitive nano‐covalent organic polymer to co‐deliver a PD‐L1 modulator, which suppresses tumor immune escape via PD‐L1 downregulation and synergistically enhances antitumor efficacy.^[^ ^116^ ^]^ In summary, stimuli‐responsive delivery technology enhances tumor immunotherapy efficacy while minimizing systemic adverse effects induced by immune checkpoint blockade.

#### 3.3.3 TME‐Responsive Nanomaterials Target Metabolism to Overcome Immunosuppressive TME

Aberrant metabolic reprogramming in the TME, characterized by hypoxia, lactate accumulation, adenosine excess, tryptophan/glutamine catabolism, dysregulated macromolecule synthesis, and altered redox homeostasis, not only sustains tumor cell survival but also directly suppresses effector immune cell function, thereby driving immune evasion.^[^ ^117^ ^]^ Precise targeting of TME metabolic aberrations to remodel the immunosuppressive landscape enhances immunotherapy efficacy. The spatiotemporally controlled delivery of metabolic modulators via TME‐responsive nanomaterials represents a novel approach to potentiate synergistic efficacy through metabolic‐immunotherapy integration.

Song Yujun's team developed a ROS‐driven gene‐editing nanomotor (RDN@PL) with a hemin core encapsulating CRISPR/Cas9 plasmids targeting lactate dehydrogenase A (LDHA). Following cellular internalization, heme oxygenase‐1 (HO‐1) degrades RDN@PL to release carbon monoxide (CO) and plasmids. LDHA knockout suppresses glycolysis in tumor cells, thereby reducing their consumption of glucose in the TME. This alleviation of metabolic competition makes glucose more available for cytotoxic T cells, which is essential for their activation, proliferation, and effector functions. Consequently, this metabolic remodeling reverses the immunosuppressive microenvironment and promotes a robust anti‐tumor immune response. Meanwhile, CO elevates mitochondrial ROS levels, triggering metabolic disruption and immunoenhancement (Figure 4g).^[^ ^118^ ^]^ In a parallel approach, an engineered microenvironment‐responsive nanotherapeutic co‐targets glycolytic and mitochondrial energy metabolism pathways in both tumor cells and TAMs to modulate metabolic homeostasis. When combined with radiotherapy, this system reprograms the immunosuppressive microenvironment by reversing radiation‐induced M2 macrophage polarization, thereby potentiating antitumor immunity. The design employs PEGylated liposomes as a dual‐delivery system for mannose and levamisole hydrochloride, enabling simultaneous inhibition of glycolysis and mitochondrial metabolism.^[^ ^119^ ^]^ Notably, therapy resistance drives prostate cancer progression to refractory disease, a state frequently exhibiting dysregulated lipid metabolism. Emerging studies demonstrate that modulating tumor lipid metabolism further enhances immunotherapeutic efficacy in such contexts.^[^ ^120^ ^]^

### 3.4 Effector Phase: Amplifying Effector Immune Cell Cytotoxicity in the Tumor Milieu

Following activation, immune cells mediate antigen‐specific recognition and targeted killing to eliminate tumor cells or pathogens. This process constitutes the pivotal determinant of therapeutic efficacy. During this phase, the TME establishes multifaceted defensive mechanisms through physical barriers, metabolic stress, and immunosuppressive networks, leading to attenuated cytotoxic function of effector cells and even inducing terminal exhaustion or cell apoptosis.^[^ ^121^ ^]^ Deciphering the dynamic immune editing processes within this stage and developing spatiotemporally precise intervention strategies are critical for overcoming the bottlenecks of immune therapy. TME‐responsive nanomedicines enhance therapeutic response by improving T cell infiltration through increased vascular extravasation and stromal penetration, while sustaining metabolic plasticity and stemness‐like properties to prolong durable cytotoxic activity.

#### 3.4.1 TME‐Responsive Nanomaterials Facilitate Effector Immune Cell Infiltration

The physical barriers and immunosuppressive properties of the TME are key factors impeding immune cell infiltration, leading to insufficient density of effector immune cells within tumor tissues and severely compromising the efficacy of immunotherapy.^[^ ^5^, ^122^ ^]^ Microenvironment‐responsive NPs can significantly enhance the recruitment and infiltration of effector immune cells into tumor tissues by targeting and modulating both the physical structure of the TME and its immunosuppressive molecules. This strategy remodels the physical‐immunological barriers, providing navigational guidance and barrier clearance support for immune cell infiltration into the deep tumor regions. It represents a promising strategy for improving the response rates of tumor immunotherapy.

**Figure 5:**
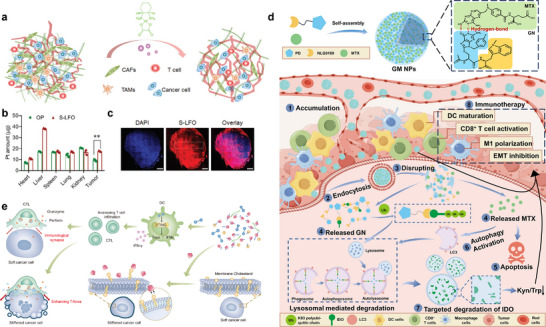
The examples of TME‐responsive nanomaterials amplifying effector immune cell cytotoxicity. a) Schematic illustration of the S‐LFO‐mediated remodeling of intratumor physical barriers to potentiate CTL infiltration in tumors. b) Quantified distribution of S‐LFO and free OP in the major organs by measuring Pt concentration via ICP‐MS analysis. The data are means ± SD, n = 3. c) Intratumor distribution of Dil‐labeled S‐LFO in the whole tumor tissues as detected via CLSM, scale bar, 1 mm. Reproduced with permission.^[^ ^123^ ^]^ Copyright 2022, Wiley. d) Schematic illustration of supramolecular artificial Nano‐AUTACs for synergistic tumor therapy. Reproduced with permission.^[^ ^126^ ^]^ Copyright 2024, AAAS. e) Schematic of cancer‐cell stiffening using NPs boosts T force‐mediated killing for anticancer STING immunotherapy. NPs generate robust anti‐tumor immunity by synergistically enhancing T‐cell intratumoral infiltration and T‐cell force, thereby potentiating T‐cell cytotoxicity. Reproduced with permission.^[^ ^129^ ^]^ Copyright 2025, Springer Nature.

Analysis of clinical samples from breast and colorectal cancer patients revealed widespread deposition of various ECM components but a severe deficiency in CD8⁺ T cell infiltration at tumor sites. Based on this observation, Zhang et al. designed and synthesized an intracellularly reduction‐responsive nitric oxide (NO) donor‐oxaliplatin prodrug (FO). They then constructed a highly efficient, biomimetic lipoprotein system (S‐LFO) designed to target diverse stromal cell populations within the tumor. Treatment with S‐LFO significantly promoted tumor vascular normalization, perfusion capacity, and vessel density, while reducing the proportions of TAMs and CAFs. Furthermore, S‐LFO depleted major ECM components, thereby smoothing the path for enhanced intratumoral infiltration of CTLs. Corresponding experimental results demonstrated that S‐LFO significantly increased both the number of CD3⁺CD8⁺ T cells and the proportion expressing IFN‐γ and Granzyme B within tumors. It also markedly enhanced the infiltration and diffusion capacity of CD8⁺ T cells into the 4T1‐GFP cancer cell regions deep inside tumors. Consequently, S‐LFO elicited potent immune‐mediated tumor killing across multiple tumor models. This strategy provides a novel approach for boosting CTL infiltration to enhance the efficacy of immunotherapy (**Figure** **5**a–c).^[^ ^123^ ^]^ Beyond stromal remodeling that facilitates T cell infiltration, NP‐mediated delivery and TME‐responsive release of chemokines can locally enrich chemokine gradients, thereby recruiting T cells for directional migration and enhancing their intratumoral infiltration. Employing a multicomponent cooperative self‐assembly approach, researchers engineered a TME‐responsive nano‐immunomodulator. This nanosystem responsively releases C–C chemokine ligand 5 (CCL5), which reconstructs the disrupted immune chemokine gradient, thereby enhancing the recruitment of CAR‐T cells and their infiltration into deep tissue regions. Synergistically coupled with the reversal of TME immunosuppression mediated by IDO within the system, this strategy creates a favorable microenvironment for CAR‐T cells to exert their cytotoxic functions.^[^ ^124^ ^]^

#### 3.4.2 TME‐Responsive Nanomaterials Sustain Effector T Cell Activity

During the effector phase of immunotherapy, activated T cells migrate to tumor sites via chemokine guidance, recognize tumor‐associated antigens presented by MHC class I molecules, and subsequently induce tumor cell apoptosis through perforin/granzyme release or IFN‐γ secretion.^[^ ^125^ ^]^ Beyond enhancing T cell infiltration, maintaining and augmenting the activity of these effector T cells is paramount for achieving potent tumor elimination. However, the immunosuppressive TME drives T cell exhaustion, substantially compromising their antitumor efficacy. TME‐responsive nanomaterials address this challenge by precisely recognizing TME hallmarks to achieve spatiotemporally controlled release of therapeutic agents at tumor loci. This strategy potentiates effector T cell function through multiple modulation of signal transduction pathways and cellular survival, thereby advancing novel therapeutic paradigms for enhanced cancer immunotherapy.

Integrating this emerging technology, we engineered a TME‐responsive nanosystem to potentiate T‐cell activity, thereby significantly enhancing antitumor immunity. Inspired by the guanine scaffold of autophagy‐targeting chimeras (AUTACs), a revolutionary targeted degradation platform developed in recent years, we synthesized a supramolecular artificial AUTAC nanomedicine (designated GM NPs) via non‐covalent interactions between an IDO degrader and the nucleoside analog methotrexate (MTX). The acidic TME disrupts supramolecular interactions to release MTX, which concurrently activates DCs and induces autophagy. Crucially, the triggered autophagy promotes degradation of immunosuppressive IDO by liberated GN moieties, ultimately enhancing effector T‐cell function while suppressing tumor growth and metastasis. This study pioneers a unique strategy leveraging AUTAC‐inspired nanotechnology to reinvigorate T cells for advanced cancer immunotherapy (Figure 5d).^[^ ^126^ ^]^ Modulating cancer cell properties represents a potent complementary strategy to enhance T‐cell‐mediated tumor killing and amplify immunotherapeutic efficacy. T cells generate mechanical force to enable perforin‐mediated membrane pore formation, boosting tumor killing.^[^ ^127^ ^]^ Yet tumors resist by softening membranes via cholesterol/F‐actin alterations, making cholesterol modulation a key therapeutic strategy.^[^ ^128^ ^]^ Leveraging the reductive TME, Yang et al. engineered a stimuli‐responsive nanoplatform for controlled release of macrocyclic methyl‐β‐cyclodextrin (MβCD), which depletes membrane cholesterol to counteract cancer cell mechanical softness, thereby potentiating CTL‐mediated tumor killing. This system demonstrated robust tumor eradication and long‐term immunological memory in female tumor‐bearing murine models (Figure 5e).^[^ ^129^ ^]^ Stimuli‐responsive cytokine release leveraging the TME significantly sustains T‐cell activity. IL‐15 critically promotes survival and proliferation of natural killer (NK) cells and CTLs.^[^ ^130^ ^]^ Building on this, Li et al. engineered bioengineered vesicles featuring a doxorubicin‐loaded ferritin core functionalized with acid‐sensitive, membrane‐anchored IL‐15/IL‐15Rα complexes. Spatiotemporally released IL‐15 complexes specifically engage IL‐15 receptor β/γc dimers, thereby expanding and activating CTLs/NK cells to induce polyfunctional cytotoxic immunity.^[^ ^131^ ^]^

Overall, strategies targeting metabolic reprogramming, biomechanical reversal, and cytokine signaling activation disrupt tumor immune editing barriers to sustain and enhance effector T‐cell function through multidimensional mechanisms. Such microenvironment‐responsive platforms establish an immunocyte‐potentiation paradigm that overcomes tumor immunotherapy resistance, thereby advancing intelligent stimuli‐responsive immunotherapy technologies.

## 4 Clinical Translation Landscape

The clinical translation of TME‐responsive nanomedicines for cancer immunotherapy remains at a nascent stage, with most projects focused on preclinical research or early‐stage clinical trials (Phase I/II). **Table** **2** lists the selected clinical trials involving TME‐responsive nanomaterials. Core strategies employ nanocarriers to deliver immunomodulators including IL‐12 mRNA (JCXH‐211), STING agonists (ONM‐501), and TLR7/8 agonists (TransCon, NCT04799054), with spatiotemporally controlled release triggered by TME‐specific stimuli such as acidic pH (CriPec® Docetaxel, NCT02442531; CPC 634), enzymatic overexpression (BIND‐014, NCT01812746), or dysregulated metabolites (CNSI‐Fe(II), NCT06048367). While preclinical and early clinical data demonstrate improved targeting and reduced systemic toxicity, critical barriers persist: 1) TME heterogeneity compromises activation precision (e.g., ATP gradients), 2) manufacturing complexity impedes GMP scalability (core‐crosslinked micelles in CPC 634), and 3) immune modulation requires finer spatiotemporal control (evidenced by ONM‐501/cemiplimab combos). Future advancement demands innovations in material design fidelity, deeper interrogation of tumor‐immune crosstalk, and coordinated multidisciplinary efforts to overcome translational bottlenecks. Despite these challenges, the expanding clinical pipeline underscores their transformative potential in redefining immunotherapy paradigms.

**Table 2:** Selected clinical trials involving TME‐responsive nanomaterials.

Trial	Phase	Start year	Status	Response	Description	Efficacy	Cancer	Refs.
JCXH‐211 NCT05727839	Phase I	2023	Recruiting	Enhanced translatability in immunosup‐pressed TME	A self‐replicating ribonucleic acid encoding human IL‐12 encapsulated in lipid NPs	Good safety profile, antitumor activities were observed in patients. Significant increases of T and NK cell infiltration	Malignant solid tumors	[132]
Carbon Nanoparticle‐Loaded Iron [CNSI‐Fe(II)] NCT06048367	Phase I	2022	Completed	Oxidation	Fe^2^⁺ is oxidized to Fe^3+^ within tumor cells, generating a large amount of ROS, which leads to tumor cell apoptosis	Safety, tolerability, and demonstration of initial efficacy in patients	Advanced solid tumors	[133]
ONM‐501 NCT06022029	Phase I	2023	Recruiting	pH	A pH‐sensitive cGAMP‐conjugated PC7A polymer designed to activate STING signaling, with or without cemiplimab	Demonstrated safety in rats and crab‐eating monkeys. The clinical trial is underway	Advanced solid tumors and lymphomas	[134]
TranscendIT‐101 NCT04799054	Phase I/II	2021	Active, not recruiting	pH	TransCon TLR7/8 agonist, comprising resiquimod conjugated to PEG microspheres via a cleavable pH‐sensitive linker	Enable sustained TME release of resiquimod and potent antitumor activity, both alone and with immunotherapy	Locally advanced or metastatic solid tumors	[135]
CPC 634 (CriPec®Docetamine) NCT03742713	Phase II	2018	Completed	pH	CPC 634 is a 65 nm polymeric micelle that releases docetaxel via acid‐triggered bond	No results posted	Advanced epithelial ovarian cancer	[136]
NC‐6300 NCT03168061	Phase I/II	2017	Last known status is recruiting	pH	An epirubicin‐conjugated polymeric micelle that releases the drug in tumors via a pH‐sensitive linker	Promising anti‐tumor activity was observed in patients	Advanced solid tumors or soft tissue sarcoma	[137]
CriPec® Docetaxel NCT02442531	Phase I	2015	Completed	pH	Docetaxel is connected to a polymer carrier via an acidic‐responsive bond	Can be administered safely but showed cumulative, but reversible skin toxicity at high doses	Solid tumors	[136]
BIND‐014 (Docetaxel Nanoparticles for Injectable Suspension) NCT01812746	Phase II	2013	Completed	Enzyme	Docetaxel is bound to the carrier via a linker that can be cleaved by proteases	Active and well tolerated in patients. Antitumor activity may be related to PSMA expression levels	Prostate cancer	[138]

## 5 Challenges and Future Perspectives

TME‐responsive nanomedicines offer transformative opportunities for enhancing cancer immunotherapy. Their paramount advantage lies in spatiotemporally precise modulation of the cancer‐immunity cycle. By incorporating stimulus‐responsive elements, these nanoplatforms achieve tumor‐specific activation of therapeutic payloads within the TME. During the initiation phase, they induce ICD to accelerate antigen release and cross‐presentation. In the activation phase, they reverse immunosuppression by reprogramming immunosuppressive cells and blocking checkpoint pathways, thereby potentiating T cell priming. At the effector phase, they enhance T cell infiltration and sustain cytotoxic activity through metabolic reprogramming. Furthermore, these systems demonstrate significant synergistic efficacy when combined with existing immunomodulatory agents.^[^ ^139^ ^]^

Despite significant advances in these responsive delivery systems across various cancer immunotherapy strategies, clinical translation remains challenging. Tumor heterogeneity constitutes a fundamental barrier to precise regulation. The expression of stimulus‐triggering mechanisms varies across tumor sites, and this spatiotemporal dynamic variation in the TME compromises the reliability of responsive strategies.^[^ ^140^ ^]^ Second, the most critical challenge lies in the inability of most currently used models to replicate authentic TME features. Compared to primary tumors in cancer patients, these models fail to fully recapitulate the immunological complexity of patients.^[^ ^141^ ^]^ For instance, the widely studied patient‐derived xenograft (PDX) models lack human stromal components.^[^ ^142^ ^]^ These discrepancies lead to clinical failure of TME‐responsive materials despite preclinical success. Furthermore, most synthetic biomaterials developed in laboratory settings are difficult to scale up for manufacturing. Batch‐to‐batch variations exist in multi‐stimuli‐responsive NPs, and complex synthesis processes cause dramatic cost increases.^[^ ^15^ ^]^ Additionally, nano‐bio interface complexities may shield targeting ligands and deactivate responsive elements, eliciting off‐target effects.^[^ ^143^ ^]^ These challenges collectively result in a low clinical translation rate for current TME‐responsive nanomedicines.

Refinements in the preclinical discovery, optimization, and validation of potential novel therapeutics may address various barriers to the successful translation of emerging nano‐immunotherapies. With the advent of multi‐omics sequencing, our deepened understanding of the molecular landscape enables comprehensive analysis of tumors and the TME, partially explaining therapy resistance arising from subtle differences. Building upon these advanced technologies, each tumor and its associated TME can be conceptualized as a unique physiological niche in the future. Comprehensive characterization should be conducted to achieve a detailed understanding of each niche, enabling the design of tailored immunotherapies to generate niche‐specific antitumor immunity.^[^ ^144^ ^]^ Critically, priority should be given to developing high‐fidelity preclinical models such as patient‐derived organoids and 3D immune cell co‐culture systems to better simulate stromal‐immune interaction features within the TME, thereby validating the spatiotemporal release kinetics of novel nanocarriers and the immunomodulatory efficacy of their therapeutic payloads.^[^ ^145^ ^]^ During material design, scalability must be thoroughly considered. Responsive delivery systems based on FDA‐approved materials should be designed for easy scale‐up to enable high‐throughput production, while enhancing reproducibility and reducing batch‐to‐batch variability. For nano‐bio interface constraints, collaborative biomimetic camouflage and intelligent interface engineering could be explored to minimize off‐target effects.^[^ ^146^ ^]^ Undoubtedly, the biosafety of nanomedicines remains the prerequisite for their biomedical application and clinical translation. The safety of designed nanomedicines during immunotherapy should be systematically investigated to assess potential host tissue damage or immune dysfunction over the long term.

Nanomaterials enable precise tumor targeting and dynamic adaptation to the TME, facilitating either prolonged release of immunotherapeutic agents or direct alteration of the TME itself. TME‐responsive intelligent biomaterials have achieved substantial success in spatiotemporally controlling antitumor immune responses and enhancing cancer immunotherapy efficacy. By integrating stimulus‐responsive elements, these systems precisely intervene in the initiation, activation, and effector phases of the cancer‐immunity cycle. This includes critical processes such as enhancing DC antigen presentation, reprogramming immunosuppressive microenvironments, and potentiating T cell cytotoxicity. Successful clinical translation urgently requires deep collaboration across materials science, immunology, and clinical medicine to address fundamental challenges including quantifying TME heterogeneity, achieving precise nano‐bio interface regulation, and establishing standardized production protocols, thereby bridging the bench‐to‐bedside gap.

## Conflict of Interest

The authors declare no conflict of interest.
